# Data for chicken semen proteome and label free quantitative analyses displaying sperm quality biomarkers

**DOI:** 10.1016/j.dib.2014.08.008

**Published:** 2014-09-21

**Authors:** Valérie Labas, Isabelle Grasseau, Karine Cahier, Audrey Gargaros, Grégoire Harichaux, Ana-Paula Teixeira-Gomes, Sabine Alves, Marie Bourin, Nadine Gérard, Elisabeth Blesbois

**Affiliations:** a INRA, UMR85 Physiologie de la Reproduction et des Comportements, F-37380 Nouzilly, France; b CNRS, UMR 7247, F-37380 Nouzilly, France; c Université François Rabelais de Tours, F-37000 Tours, France; d IFCE, Institut Français du Cheval et de l׳Equitation, F-37380 Nouzilly, France; e INRA, Plate-forme d׳Analyse Intégrative des Biomolécules, Laboratoire de Spectrométrie de Masse, F-37380 Nouzilly, France; f INRA, UMR1282 Infectiologie et Santé Publique, F-37380 Nouzilly, France; g Université François Rabelais de Tours, UMR1282 Infectiologie et Santé Publique, F-37000 Tours, France

**Keywords:** Chicken (*Gallus gallus*), Spermatozoa, Seminal plasma, Proteome, Fertility

## Abstract

Understanding of biology of the avian male gamete is essential to improve the conservation of genetic resources and performances in farming. In this study, the semen proteome of the main domestic avian species (*Gallus gallus*) and evaluation of the molecular phenotype related to sperm quality were investigated using GeLC–MS/MS approach and label-free quantitative proteomic based on Spectral Counting (SC) and extracted ion chromatograms (XIC) methods. Here we describe in details the peptide/protein inventory of chicken ejaculated spermatozoa (SPZ) and seminal plasma (SP). We also show differential analyses of chicken semen (SPZ and corresponding SP) from 11 males demonstrating different levels of fertilizing capacity and sperm motility. The interpretation and description of these data can be found in a research article published by Labas and colleagues in the Journal of Proteomics in 2014 [1]. This is a new resource for exploring the molecular mechanisms involved in fertilizing capacity and to reveal new sets of fertility biomarkers.

**Table:** 

**Specifications table**	
Subject area	*Male gamete proteins and reproductive capacity*
More specific subject area	*Chicken (Gallus gallus) ejaculated spermatozoa and seminal plasma protein content and semen quality*
Type of data	Raw and processed/analyzed mass spectrometry data obtained by nanoliquid chromatography combined to high resolution tandem mass spectrometry,.xls tables with identified/validated and quantified proteins
How data was acquired	Experiments performed on a LTQ Orbitrap Velos Mass Spectrometer (Thermo Fisher Scientific, Bremen, Germany) coupled to an Ultimate^®^ 3000 RSLC Ultra High Pressure Liquid Chromatographer (Dionex, Amsterdam, The Netherlands)
Data format	Raw data: raw.mzml
	Processed and analyzed data using Mascot Search engine:.dat
Experimental factors	Sub/infertile and fertile chickens
Experimental features	Ejaculated spermatozoa and seminal plasma proteome (*Gallus gallus*) of experimental lines D+/D− and evaluation of the molecular phenotype related to sperm quality
Data source location	N/A
Data accessibility	Data are present here with this paper and the MS proteomics data were deposited to the ProteomeXchange Consortium (http://proteomecentral.proteomexchange.org) via the PRIDE partner repository with the dataset identifiers PXD000287 and PXD001254.
	ftp://ftp.pride.ebi.ac.uk/pride/data/archive/2014/08/PXD000287
	ftp://ftp.pride.ebi.ac.uk/pride/data/archive/2014/08/PXD001254

## Value of the data

• First description of peptidome/proteome of chicken ejaculated spermatozoa.
• First description of peptidome/proteome of chicken seminal plasma.
• Semen proteomic phenotyping at an individual level from 11 males.
• First proteome comparisons of sub/infertile and fertile chickens to characterize biomarkers related to fertility.

## 1 Data, experimental design, materials and methods

We describe here a unique dataset composed of qualitative proteomic analysis of chicken spermatozoa and seminal plasma and quantitative proteomic analyses related to semen quality. The present study identified a large number of proteins that have never previously been described in *Gallus gallus*. Furthermore, label free quantitative proteomic analyses combined with physiological tests allowed phenotyping semen at the individual level and characterization of new peptides and proteins that are biomarker candidates of fertility.

## 2 Semen collection and physiological tests

Eleven adult males of a standardized genetic origin [2] housed in the INRA PEAT experimental unit (Nouzilly, France) were collected twice a week. Sperm concentrations were determined [3] and individual ejaculates were diluted 1:1 in Beltsville Poultry Semen Extender (BPSE, [4]) before mass motility analysis or female insemination. Mass motility was measured as a subjective evaluation of the speed of movement of a group of sperm as previously described [5]. Fertility (% fertile/incubated eggs) was measured after individual single intravaginal artificial insemination (mean of ten females per male).

## 3 Preparation of sperm and seminal plasma for proteomic analysis

Semen (3 ejaculated samples) was collected from the 11 male chickens. Spermatozoa (SPZ) were separated from seminal plasma (SP) by centrifugation. SPZ were frozen-thawed with glycerol cryoprotectant as previously described [6]. At thawing, glycerol was removed from the semen by successive dilutions (1:1) with BPSE supplemented with a Protease Inhibitor Cocktail (cOmplete, Mini, EDTA-free, Roche Applied Science) and centrifugation. SPZ proteins were extracted using following buffer (150 mM NaCl, 10 mM TrisHCl pH7.4, 1 mM EDTA, 1 mM EGTA, 1% Triton X100, 0.5% NP40, distilled H_2_O qs 100 mL) supplemented with anti-proteases. Samples were strongly shaken at intervals for 30 min at 4 °C. The mix was centrifuged for 20 min at 13,000*g* at 4 °C. The protein concentrations of supernatants containing sperm proteins were determined using a Protein DC Assay (Bio-Rad, Marnes-la-Coquette, France).

### 3.1 GeLC–MS/MS analyses

For exhaustive inventory, 25 µg of proteins from pools of SP and SPZ collected from the 11 males were fractionated by SDS-PAGE (4–20%, minigel) and stained by Coomassie Blue. The whole lane was sectioned into 40 bands.

For differential analyses, 25 µg of SP and SPZ protein samples for each chicken were included in SDS-PAGE (10%, minigel) without fractionation and stained by Coomassie Blue. One band was cut.

Each gel slice was in-gel digested using trypsin as previously described [1]. The extracted peptides were analyzed by on-line nanoflow liquid chromatography–tandem mass spectrometry (nanoLC–MS/MS) using a dual linear ion trap Fourier Transform Mass Spectrometer (FT-MS) LTQ Orbitrap Velos (Thermo Fisher Scientific, Bremen, Germany) coupled to an Ultimate^®^ 3000 RSLC Ultra High Pressure Liquid Chromatographer (Dionex, Amsterdam, The Netherlands) as previously described [1]. The mass spectrometer was operated in positive mode in data-dependent mode with high resolution (*R*=60,000) full scan MS spectra (profile mode) and low-resolution CID-MS/MS (centroid mode). In the scan range of *m*/*z* 300–1800, the 20 most intense peptide ions with charge states ≥2 were fragmented by CID. Polydimethylcyclosiloxane (*m*/*z*, 445.1200025) ions were used as lock mass for internal calibration. Each band was analyzed by nanoLC–MS/MS with one and four replicates, for qualitative and quantitative analysis respectively.

### 3.2 Protein identification and validation

All raw data files were converted to Mascot Generic Format (MGF) with Proteome Discoverer 1.2 software (Thermo Fisher Scientific). All MS/MS data were analyzed using MASCOT 2.3 search engine (Matrix Science) against the chordata section of a locally maintained copy of nrNCBI (19922528 sequences, download 08/21/2012). Enzyme specificity was set to trypsin with two missed cleavages using carbamidomethylcysteine, oxidation of methionine and N-terminal protein acetylation as variable modifications. The mass tolerance was set at 5 ppm for parent and 0.8 Da for fragment ions. The MS proteomics data (raw.mzml and search results.dat were deposited with the ProteomeXchange Consortium (http://proteomecentral.proteomexchange.org) via the PRIDE partner repository [7,8] with the dataset identifiers PXD000287 and PDX001254. In order to validate identified proteins, data search results (.dat files) were incorporated in Scaffold 3.4 software (Proteome Software).

### 3.3 Spermatozoa and seminal plasma proteomes

Qualitative analysis using GeLC–MS/MS strategy (SDS-PAGE fractionation with 40 bands combined to nanoLC–MS/MS after in-gel digestion) generated a total of 78,988 and 33,540 fragmentation spectra in SPZ and SP protein samples, respectively (pools of the 11 chickens). A total of 1165 different proteins were identified in semen and validated with≥95% confidence as specified by the Peptide and Protein Prophet algorithms [9,10], with at least two distinct peptides and a false discovery rate (FDR)<1%. In total, we cataloged 822 and 607 non-redundant proteins for SPZ and SP, respectively. A total of 264 proteins were common between SPZ and SP while 558 proteins were unique to SPZ and 343 proteins were unique to SP. Details of all identified SPZ and SP proteins are provided in Supplementary Table 1. Here, common proteins and specific proteins are highlighted among these 1165 proteins. For each protein, we present the protein description, the GI Accession number, gene name, theoretical molecular weight and scaffold software results for the protein identification probability, number of assigned spectra, number of unique peptides or spectra and the % of sequence coverage.

### 3.4 Label free quantitative proteomic analyses and relationship with sperm fertilizing ability study

Quantitative analyses were performed on SPZ and SP samples from the 11 males. Protein samples included in polyacrylamide gel without fractionation were analyzed by nanoLC–MS/MS after in-gel digestion, *n*=4 technical replicates. A total of 314 and 144 unique proteins were identified for SPZ and SP, respectively (FDR<1%).

For global comparison of the 11 males, GeLC–MS/MS analyses were combined to a label free quantitative method based on Spectral Counting (SC) using Scaffold 3 Q+ software. For protein quantification without ambiguity, we considered proteins with more than two peptides identified and ignored all peptide shared between protein groups (family). After ANOVA, 187 and 124 unique proteins were characterized differentially (*p*<0.05) between the 11 animals for SPZ and SP, respectively. Details of differential and quantitative results using SC method obtained from global comparison of the eleven chickens are provided in Supplementary Table 2. In the table, for each protein, we present the protein description, the GI Accession number, gene name, theoretical molecular weight and scaffold software results for ANOVA, the protein identification probability, number of assigned spectra, number of unique peptides or spectra and the % of sequence coverage, unweighted spectrum count and quantitative values.

In order to identify proteins linked to fertilizing ability, GeLC–MS/MS analyses were combined to two different label free quantitative methods: Spectral Counting (SC) using Scaffold 3 Q+ software and eXtracted Ion Chromatogram peptide pattern (XIC) using SIEVE v 1.3 software (Thermo Fisher Scientific). Only proteins quantified by both SC and XIC MS-based quantitative methods were retained. Comparisons were performed to characterize changes between one highly fertile male (chicken 11) and one infertile (chicken 6), as well as two highly fertile males (5 and 11) and the two least fertile males (6 and 8). SC quantifications were performed as previously described using normalized spectral counts on distinct proteins while XIC quantifications were carried out on normalized XIC values between 2 sample groups, frame-by-frame. The XIC results were filtered with protein normalized ratios <0.5 and >2, with Mascot ion scores >20. For the two quantitative methods, differences were considered statistically significant at *p*<0.05. For XIC quantification, the repeatability linked to the nanoLC–MS/MS process was evaluated by a normalized standard deviation (NStdDev). NStdDev was calculated using normalized area peak of autodigest tryptic peptides of the 4 replicates for each sample. Mean NStdDev values did not exceed 26 and 31% respectively for sperm and fluid analyses. Furthermore, immunodetection on superoxide dismutase [Cu–Zn] protein (SOD) confirm the variable abundance observed from MS analyses.

The combined quantification showed that 31 and 40 SPZ proteins plus 24 and 48 SP proteins were differential for the two comparisons, respectively. Details of differential and quantitative results using SC and XIC methods in relation to fertility are provided in Supplementary material, Table S1. The differential proteins quantified by both SC and XIC pattern are listed for SPZ and SP, respectively, according to *t*-tests between one highly fertile male (chicken 11) and one infertile (chicken 6), as well as those of two most fertile males (5 and 11) and the two least fertile males (6 and 8). We present the protein description, GI Accession number, gene name, theoretical molecular weight and scaffold software results for the *p* value *t*-test for each protein, with the protein identification probability, number of assigned spectra, number of unique peptides or spectra and the % of sequence coverage, unweighted spectrum count and quantitative values. The SIEVE software results are also presented with the ratio, number of peptides and frames used for the quantification and the *p* value.

## Conflict of interest

The authors declare that they have no conflicts of interest.
